# CBT and graded exercise therapy studies have proven that ME/CFS and long COVID are physical diseases, yet no one is aware of that

**DOI:** 10.3389/fnhum.2025.1495050

**Published:** 2025-01-29

**Authors:** Mark Vink, Alexandra Vink-Niese

**Affiliations:** ^1^Private Practitioner, Amsterdam, Netherlands; ^2^Independent Researcher, Hannover, Germany

**Keywords:** CBT, chronic fatigue syndrome, CFS, cognitive behavioral therapy, COVID-19, long COVID, ME, ME/CFS

## Introduction

The cognitive behavioral model (CBmodel) (Surawy et al., 1995; Vercoulen et al., 1998) has dominated the world of myalgic encephalomyelitis/chronic fatigue syndrome (ME/CFS) since the 1990s. According to this model, a belief in an organic illness, known as dysfunctional beliefs, stops ME/CFS patients engaging in normal activities, resulting in avoidance behavior and deconditioning. The deconditioning then leads to further avoidance behavior and more deconditioning. According to the CBmodel, symptoms of ME/CFS are caused by deconditioning and not by an underlying illness. Cognitive behavioral therapy with graded activity (CBTplus) and graded exercise therapy (GET) were designed to reverse the dysfunctional beliefs, the avoidance behavior and the deconditioning and lead to recovery. However, an extensive review of the literature found that CBTplus and GET do not restore the ability to work (Vink and Vink-Niese, 2024b). Additionally, there are now many papers documenting complex disruptions to the body's physiology in ME/CFS, particularly involving immunological and inflammatory pathways, autonomic and neurological dysfunction, abnormalities in the cellular energy production and the gene expression (Committee on the Diagnostic Criteria for Myalgic Encephalomyelitis/Chronic Fatigue Syndrome, 2015; Liu et al., 2024; Missailidis et al., 2019).

## Discussion

### 1 Evidence from the studies

As many runners know, if you are a beginner and you start exercising three times a week, you can run half a marathon in 12 weeks. In a healthy sedentary person who does not do physical exercise or work, that will take around 12–24 weeks (The Runner's World Editors, 2023).

The assumption of the CBmodel is that ME/CFS patients are simply deconditioned. This means that they should respond in the same way to exercise as healthy sedentary controls who are deconditioned because they don't do exercise or physical work and activities. Let's keep that in mind and have a look at the largest CBTplus and GET trial for ME/CFS, the PACE trial (*n* = 641) (White et al., 2011), and its GET group, in particular. The 160 participants in that group were exercising 5 days a week for up to 30 min per day for 24 weeks. If there would be no underlying disease, and patients were merely deconditioned, then such an exercise regime would lead to a very substantial improvement in fitness. However, fitness did not improve (Chalder et al., 2015). The same thing was seen in the CBTplus group. Consequently, something in the body of these patients, i.e. an underlying disease, in this case ME/CFS, was preventing that. It also means that patients were already exercising at their maximum when they joined the study, which disproves the assumption that patients were exercising less than they could due to dysfunctional beliefs.

Three Dutch studies (Prins et al., 2001; Stulemeijer et al., 2005; Knoop et al., 2008) showed the following: 8 months of CBTplus in adults, 5 months of CBTplus in adolescents and at least 16 weeks of guided self-instructions in adults, based on CBTplus, did not lead to an objective improvement of activity (actometer) (Wiborg et al., 2010). A 12-week programme of GET (Moss-Morris et al., 2005), an 18-week programme combining CBTplus and GET in the more severely affected (Wearden and Emsley, 2013) and the evaluation of the efficacy of CBTplus and GET in the Belgium reference centers (Stordeur et al., 2008) showed no objective improvement in fitness, according to VO_2_peak, a timed step test, which “strongly and reliably predicts the maximal aerobic capacity VO_2_max” (Petrella et al., 2001, p. 630) and VO_2_peak or VO_2_max, respectively. This is not only important for ME/CFS patients, but also for those with long COVID because 51% of them fulfill ME/CFS criteria according to a systematic review (Dehlia and Guthridge, 2024). There are between 65 and 100 million long COVID patients based on best estimates (Davis et al., 2023; Perumal et al., 2023). This means that there are between 33 and 51 million extra patients with ME/CFS on top of the estimated 17–24 million patients that were already there (Lim et al., 2020) and more and more researchers are getting interested to investigate the parallels between ME/CFS and long COVID (Annesley et al., 2024; Jason et al., 2023; Komaroff and Lipkin, 2023; Proal and VanElzakker, 2021; Wong and Weitzer, 2021).

In all the aforementioned CBTplus and GET studies, one would have expected a (very) substantial increase in activity/fitness but this didn't happen because an underlying illness was preventing that, just like what was found by the PACE trial. Consequently, all those studies proved that ME/CFS is a physical disease and that ME/CFS patients do not suffer from dysfunctional beliefs. This also invalidates the CBmodel. Additionally, this confirms the findings from Sunnquist and Jason (2018) who re-examined the CBmodel, concluding that their findings were inconsistent with it. Two reviews also concluded that the psychosomatic view on ME/CFS of the CBmodel is inconsistent with the current evidence (Geraghty et al., 2019; Thoma et al., 2024).

Finally, the ReCOVer study, based on the CBmodel, found that 16 weeks of CBTplus did not lead to an objective improvement of activity (actometer) in long COVID either (Kuut et al., 2023a,b). Thereby proving that long COVID is a physical disease and that long COVID patients do not suffer from dysfunctional beliefs either.

### 2 The cardinal symptom of ME/CFS: post-exertional malaise

The beliefs that patients have about their energy levels are not dysfunctional, because they are affected by the cardinal symptom of ME/CFS: post-exertional malaise (PEM). Many people think that post-exertional fatigue is PEM. Yet fatigue after exertion is simply a normal physiological response to exercise. PEM on the other hand is an increase in symptoms, disproportionate to the level of exertion. Its onset is often delayed for up to 72 h, it is accompanied by a loss of strength and/or loss of function, and the recovery from it is abnormally delayed (Committee on the Diagnostic Criteria for Myalgic Encephalomyelitis/Chronic Fatigue Syndrome, 2015; Ramsay, 1986; Bateman et al., 2021; Carruthers et al., 2011; Vink and Vink-Niese, 2020). All these elements are compulsory for a diagnosis of PEM which is the very reason why patients cannot increase their activity levels as desired by the CBmodel and its treatments.

A large study that compared ME/CFS (*n* = 84) with healthy sedentary controls (HCTLs; *n* = 71) by using cardiopulmonary exercise testing separated by 24 h (2-day CPET), found that unlike HCTLs, ME/CFS failed to reproduce CPET-1 measures during CPET-2 with significant declines at peak exertion (Keller et al., 2024). Other but smaller 2-day CPET studies, had found similar abnormalities (Davenport et al., 2020; Snell et al., 2013; van Campen and Visser, 2021; Keller et al., 2014; van Campen et al., 2020). Yet if patients would have been merely deconditioned, then there would not have been a difference during CPET-2 with the HCTLs.

### 3 Adherence to the treatment

Some people might say, but that is simply down to the fact that patients were not motivated to follow those treatments and they simply did not adhere to them. However, the above mentioned studies concluded that their treatments were effective which implies that patients adhered to treatment. If patients had not adhered to treatment, then those studies would have concluded, we cannot conclude anything about the efficacy of our treatments because patients did not adhere to it. Or, that patients did not adhere to the treatment because it was not effective and/or patients were negatively affected by it. Moreover, the aforementioned PACE trial (White et al., 2011) found high rates of acceptance of the treatments and of participants satisfaction; 87% (CBTplus) and 85% (GET) of participants were adequately treated, the adherence to the manual by competent therapists was very good (CBTplus) and excellent (GET), and the dropout rate was low (11%, CBTplus and 6%, GET).

Additionally, the aforementioned Belgium evaluation (*n* = 655) (Stordeur et al., 2008) concluded that patients had on average 41–62 h of CBTplus and GET, spread over 6–8 months. The dropout rate was very low (only 2.8%) because patients were “generally speaking…very motivated to follow the therapy” (Stordeur et al., 2008, p. 80). This supports the observation that ME/CFS patients are usually very motivated to engage in any treatment that might improve their often debilitating condition. At the same time, they are knowledgeable enough to pull back if they have a relapse' response to it.

An important side note is the fact that the studies claimed that their treatments were effective yet they did not consider the unreliability of subjective outcomes in non-blinded trials, or the serious flaws of their own studies included. These flaws included poorly designed control groups, relying on an unreliable fatigue instrument as a primary outcome, outcome switching, p-hacking, ignoring evidence of harms or not reporting it, etc. The studies also did not take into account that the small, short-lived subjective improvement of fatigue after CBTplus and GET, is not matched by an objective improvement of physical fitness or employment and illness benefit status (Vink and Vink-Niese, 2024b; Ahmed et al., 2020; Geraghty, 2017; Marks, 2017; Twisk and Corsius, 2018; Vink and Vink-Niese, 2019b, 2024a; Wilshire, 2017; Wilshire et al., 2018). Also, that CBTplus and GET did not lead to a reduction in CFS symptom count (White et al., 2011).

The severe problems of this line of research were highlighted by a recent analysis (Vink and Vink-Niese, 2019a) of a systematic review (Kuut et al., 2024) whereby the systematic reviewers failed to inform the readers of their conflicts of interest. For example, at least one of them was involved in all of the eight studies in their review. Conducting a review in this manner and not informing the readers, undermines the credibility of a systematic review and its conclusion. Moreover, the systematic review labeled CBTplus effective, even though according to their own results, patients were still severely disabled after treatment with CBTplus.

### 4 Why no one is aware of that

The remaining question then is, why is no one aware of the fact that CBTplus and GET studies have proven that ME/CFS and long Covid are physical diseases? Or to put it differently, why did none of these studies report this discovery? The first possible answer is because the studies were conducted by researchers who have originated and/or devoted their career to the CBmodel and the efficacy of CBTplus and/or GET for ME/CFS. The underlying assumption of that model probably is that the blueprint of treating dysfunctional cognitions with CBT works for many mental health disorders, like anxiety or depression, but it does not work for ME/CFS. As noted by Ioannidis, “investigators working in any field are likely to resist accepting that the whole field in which they have spent their careers is a ‘null field”' (Ioannidis, 2005, p. 0700).

The second possible explanation is that the studies were conducted by mainly mental health experts who are not experts in exercise physiology. Consequently, they did not see what their own results showed. In a similar manner that most of us would have thrown away the mold overgrown petri dish in the research by Dr. Alexander Fleming that led to the discovery of penicillin (Fleming, 1929). It needed someone like him to understand the meaning of it.

### 5 How the field should move forward

Progress in understanding the underlying mechanisms in ME/CFS has been hampered by small heterogeneous studies, a lack of research money in relation to the severity of the disease and psychologisation of it. What is needed are well executed, properly designed and powered studies that use the international consensus criteria (Carruthers et al., 2011) so that research populations are as homogeneous as possible. Studies should also use at least one objective primary outcome that is relevant to patients, for example, the step test, 2-day CPET or work status instead of relying on subjective outcome measures.

Research should prioritize elucidating the exact mechanism of PEM, finding effective pharmacological treatment and a reliable diagnostic test (Tyson et al., 2022). As the condition worsens, the probability of identifying biomarkers for the disease increases, making it crucial to study the severely ill (Arron et al., 2024) who are home or bed bound and make up 25% of the ME/CFS population (Pendergrast et al., 2016).

Finally, psychologists and other mental health experts should only be involved if patients need help learning how to cope with their illness, if they've got comorbid mental health problems like depression or anxiety (NICE, 2021; National Institute for Health Care Excellence, 2021). Or, if patients need help effectively implementing pacing strategies (Grande et al., 2023).

## Conclusion

Cognitive behavioral therapy and graded exercise therapy studies have proven that ME/CFS and long COVID are physical diseases. Yet no one is aware of that because many of the researchers involved in the studies have built their careers on the CBmodel and they resist accepting the true meaning of the objective outcomes of their studies because that would invalidate their model. Alternatively, the studies did not report that because most of the researchers involved are mental health experts instead of experts in exercise physiology. Consequently, they did not realize what their own studies had proven.
